# Sex-Dependent Anti-Stress Effect of an α5 Subunit Containing GABA_A_ Receptor Positive Allosteric Modulator

**DOI:** 10.3389/fphar.2016.00446

**Published:** 2016-11-22

**Authors:** Sean C. Piantadosi, Beverly J. French, Michael M. Poe, Tamara Timić, Bojan D. Marković, Mohan Pabba, Marianne L. Seney, Hyunjung Oh, Beverley A. Orser, Miroslav M. Savić, James M. Cook, Etienne Sibille

**Affiliations:** ^1^Center for Neuroscience, University of PittsburghPittsburgh, PA, USA; ^2^Department of Psychiatry, University of PittsburghPittsburgh, PA, USA; ^3^Department of Chemistry and Biochemistry, University of Wisconsin–MilwaukeeMilwaukee, WI, USA; ^4^Department of Pharmacology, University of BelgradeBelgrade, Serbia; ^5^Department of Pharmaceutical Chemistry, University of BelgradeBelgrade, Serbia; ^6^Neurobiology of Depression and Aging, Campbell Family Mental Health Research Institute, Centre for Addiction and Mental HealthToronto, ON, Canada; ^7^Department of Anesthesia–Department of Physiology, University of TorontoToronto, ON, Canada; ^8^Department of Psychiatry– Department of Pharmacology and Toxicology, University of TorontoToronto, ON, Canada

**Keywords:** Gabra5, alpha5, depression, interneurons, PAM, GABA

## Abstract

**Rationale:** Current first-line treatments for stress-related disorders such as major depressive disorder (MDD) act on monoaminergic systems and take weeks to achieve a therapeutic effect with poor response and low remission rates. Recent research has implicated the GABAergic system in the pathophysiology of depression, including deficits in interneurons targeting the dendritic compartment of cortical pyramidal cells.

**Objectives:** The present study evaluates whether SH-053-2’F-R-CH3 (denoted “α5-PAM”), a positive allosteric modulator selective for α5-subunit containing GABA_A_ receptors found predominantly on cortical pyramidal cell dendrites, has anti-stress effects.

**Methods:** Female and male C57BL6/J mice were exposed to unpredictable chronic mild stress (UCMS) and treated with α5-PAM acutely (30 min prior to assessing behavior) or chronically before being assessed behaviorally.

**Results:** Acute and chronic α5-PAM treatments produce a pattern of decreased stress-induced behaviors (denoted as “behavioral emotionality”) across various tests in female, but not in male mice. Behavioral *Z*-scores calculated across a panel of tests designed to best model the range and heterogeneity of human symptomatology confirmed that acute and chronic α5-PAM treatments consistently produce significant decreases in behavioral emotionality in several independent cohorts of females. The behavioral responses to α5-PAM could not be completely accounted for by differences in drug brain disposition between female and male mice. In mice exposed to UCMS, expression of the *Gabra5* gene was increased in the frontal cortex after acute treatment and in the hippocampus after chronic treatment with α5-PAM in females only, and these expression changes correlated with behavioral emotionality.

**Conclusion:** We showed that acute and chronic positive modulation of α5 subunit-containing GABA_A_ receptors elicit anti-stress effects in a sex-dependent manner, suggesting novel therapeutic modalities.

## Introduction

It is commonly believed that many major psychiatric conditions are stress-related disorders (Watson et al., 2016). One such disorder, major depressive disorder (MDD), is a debilitating illness characterized by several different symptom categories, including low mood, anhedonia, physiological disturbance, and increased self-focus and rumination (Ferrari et al., 2013; Northoff and Sibille, 2014). Lifetime prevalence of MDD is higher in females than in males, though factors mediating this increase are still being investigated (Seney et al., 2013). Frequently, MDD is preceded by and comorbid with other stress-associated disorders, such as generalized anxiety disorder (GAD) (Melchior et al., 2007; Moffitt et al., 2007). In fact, higher occurrence of anxiety disorders in females early in life can explain some of the increased female risk for MDD (Breslau et al., 1995). Adding to the disease burden of MDD is the relative ineffectiveness of conventional antidepressant medications (roughly 30% remission rates), most of which act via the monoaminergic system (e.g., selective serotonin reuptake inhibitors – SSRIs) (Cipriani et al., 2009; Rush, 2010). Although drugs with rapid acting antidepressant effects have shown promise in preclinical studies and effectiveness in treating severely depressed individuals (Li et al., 2010; Zarate et al., 2012a,b), no existing antidepressant drug directly addresses the pathology observed in the depressed brain, providing a potential explanation for the limited efficacy of current antidepressants.

Converging evidence has long suggested a role for the inhibitory neurotransmitter gamma-amino butyric acid (GABA) in the pathophysiology of depression (Krystal et al., 2002; Luscher et al., 2011). Studies using magnetic resonance spectroscopy show decreases in GABA levels in depression in brain regions critical for emotion regulation [dorsolateral prefrontal cortex (dlPFC), subgenual anterior cingulate cortex (sgACC)] (Sanacora et al., 1999, 2004; Bajbouj et al., 2006). At the cellular level, reduced density of calbindin-positive interneurons were reported in frontal cortex (Rajkowska et al., 2007). At the molecular level, we previously reported alterations in GABA-related gene expression in dlPFC, sgACC, and amygdala (Sibille et al., 2010; Tripp et al., 2011; Guilloux et al., 2012; Seney et al., 2014). Preclinical evidence has also implicated the GABA system in the development of stress-induced (i.e., anxiety- and depressive-like) behaviors. Mice with reduced GABA signaling exhibit increased depressive-like behavior (Earnheart et al., 2007; Shen et al., 2010). Further, chronic stress paradigms that produce anxiety- and depressive-like behavior in rodents cause GABAergic deficits in the frontal cortex, including decreases in major GABA synthesizing enzymes (Herman and Larson, 2001; Gilabert-Juan et al., 2013).

Interestingly, this alteration in GABAergic signaling in MDD subjects appears to be restricted to interneuron subtypes that project to the dendritic compartment of cortical pyramidal cells, as characterized by the expression of somatostatin (SST), neuropeptide Y (NPY) and cortistatin (CORT) neuropeptides (and that co-express calbindin); Cells targeting the perisomatic compartment, such as parvalbumin (PV)- or cholecystokinin (CCK)-expressing GABA neurons appear only sparingly affected (Sibille et al., 2010; Tripp et al., 2011; Guilloux et al., 2012). Recent work in mice has demonstrated that cortical SST cells are also affected in the unpredictable chronic mild stress (UCMS) model which induces elevated behavioral emotionality, and that UCMS-induced anxiety- and depressive-like behavior can be recapitulated in mice lacking SST (Lin and Sibille, 2015), together suggesting a causal link between low SST expression and elevated behavioral emotionality. The UCMS model has been used to assess potential drug targets for treating human depression, as it has good construct (stress precipitates the UCMS phenotype as well as MDD episodes and disrupts similar biological pathways), predictive (chronic treatment with SSRIs reverses the UCMS phenotype), and face validity (behavioral features replicated) (Edgar et al., 2011; Isingrini et al., 2012; Lin and Sibille, 2015).

Importantly, the structured anatomical distribution of GABA_A_ receptors in the cortex provides a potential means of directly addressing GABAergic dysfunction within a specific compartment. Specifically, α5-containing GABA_A_ receptors are predominantly expressed on dendritic branches of pyramidal cells where they mediate dendritic inhibition *in vitro* (Wainwright et al., 2000; Ali and Thomson, 2008), whereas α1- and α2-containing GABA_A_ receptors are found near the soma (Packer et al., 2013). This anatomical proximity suggests a functional link between dendrite-preferring SST cells and the extra-synaptic α5-containing GABA_A_ receptors that mediate tonic inhibition in the cortex (Bonin et al., 2007; Brickley and Mody, 2012). Knowing that GABA signaling is decreased in MDD and similarly in the rodent UCMS model, we set out to test whether increasing GABAergic tone specifically at the dendritic compartment may reverse UCMS-induced behaviors, a hypothesis supported by recent restriction of function experiments suggesting the α5 subunit contributes to the anxiolytic effects of diazepam, a non-selective positive allosteric modulator (PAM) of GABA_A_ receptors (Behlke et al., 2016). To directly test this hypothesis, we utilized the GABA_A_ receptor subtype-selective PAM, SH-053-2’F-R-CH3 (denoted further as α5-PAM), which has high affinity for GABA_A_ receptors containing the α5 subunit (*K*_i_ = 95.2 nM) and lower affinity for GABA_A_ receptors containing the α1- (*K*_i_ = 759.1 nM), α2- (*K*_i_ = 948.2 nM), and α3-subunits (*K*_i_ = 768.8 nM) (Savic et al., 2010). Based on the hypothesis that UCMS reduces GABAergic efficacy at the dendritic compartment, we predicted that enhancing GABA tone with α5-PAM would reverse UCMS-induced behavior in female and male mice.

Crucially, chronic stress procedures, including UCMS, disrupt multiple facets of the anxiety-/depressive-like behavioral spectrum in rodents (Willner, 2005). Likewise, MDD, as assessed by traditional clinical rating scales (e.g., Hamilton Depression Rating Scale), is typified by depressive, anxiety, and physiological/somatic symptoms that are mostly convergent on a syndrome rather than consistent within symptom dimension across time (Overall and Rhoades, 1982; Oquendo et al., 2004). The syndrome aspect of stress-induced disorders highlights the need to evaluate a battery of stress-induced behaviors in a single animal, which can then be quantified on the individual test level or as a composite “behavioral emotionality” score, analogous to an overall clinical rating score. Although this is rarely taken into consideration in rodent studies, it can be operationally accomplished using standard behavioral *Z*-scoring methods (Guilloux et al., 2011), as applied in this study. This approach has been used with success to characterize stress-associated behavioral changes in rodents (Edgar et al., 2011; Soumier and Sibille, 2014; de Sa-Calcada et al., 2015; Lin and Sibille, 2015; Quesseveur et al., 2015; Anacker et al., 2016).

## Materials and Methods

### Animals

For behavioral experiments, adult (8 weeks old) C57BL/6J mice (Jackson Laboratories, Bar Harbor, ME, USA) were housed under standard conditions with a 12/12 light/dark cycle (prior to UCMS) and *ad libitum* access to food and water in accordance the National Institutes of Health Guide for the Care and Use of Laboratory Animals, and approved by University of Pittsburgh Institutional Animal Care and Use Committee.

### Unpredictable Chronic Mild Stress (UCMS)

Mice underwent between 6 and 8 weeks of UCMS (see Supplementary Table S1 for example UCMS schedule). Animals were exposed to 2–3 mild stressors each day throughout the light/dark cycle, including wet bedding, brief restraint, forced bath, no bedding, reduced space, and predator odor over a period of several weeks (see details in Supplementary Material) (Edgar et al., 2011; Lin and Sibille, 2015). Female mice were housed 5 per cage, and male mice were housed 4 per cage in Optimice cages (Animal Care Systems Inc, Centennial, CO, USA). In experiment 3.4, UCMS was augmented with single-cage isolation housing in male mice beginning at the fourth week of UCMS until sacrifice. A timeline of experimental procedures can be found in **Figure 2A** (including timeline of the sections “ Drug Treatment” and “Behavioral Testing”).

### Drug Treatment

At the start of the third week of UCMS and continuing for between 28 and 42 days (including time for behavioral tests, see “Behavioral Testing”) until sacrifice, mice received daily i.p injections of vehicle (85% ddH_2_O, 14% propylene glycol, 1% Tween 80) or 30 mg/kg SH-053-2’F-R-CH3 (α5-PAM; synthesized in the laboratory of Dr. James M. Cook) in a volume of 10 mg/mL. All animals received a single daily injection (chronic α5-PAM treated mice received SH-053-2’F-R-CH3, acute α5-PAM and vehicle treated animals received vehicle injections) between 8:00AM and 9:00AM until the start of behavioral testing. After 3 weeks of treatment, all mice began receiving a second injection 30 min prior to behavioral testing. All animals received a second injection of either vehicle (for chronic α5-PAM treated animals and UCMS-vehicle treated animals) or α5-PAM acutely (α5-PAM acute animals). All injections were performed using ultra-fine insulin syringes (31 gauge, 6 mm; Becton, Dickinson and Company, Franklin Lakes, NJ, USA).

### Behavioral Testing

After 5 weeks of UCMS, including 3 weeks of drug treatment, animals were assessed in a battery of behavioral tests (each test separated by 48 h, UCMS continued throughout behavioral testing for a period of 1–3 weeks). These tests included: *Elevated plus maze*: under red light, animals were placed on a plus maze with two open and two closed arms (30 cm × 5 cm). Number of entries into all arms as well as the time spent in the open arms was recorded for 10 min. *Open field test*: The open field test was conducted in a 43 cm ×43 cm arena under bright (800 lux) light. Using AnyMaze software (Stoelting, Wood Dale, IL, USA), the center 50% of the arena was identified and animals were tracked for 10 min. *Novelty suppressed feeding test*: Mice were food deprived for 24 h the day before testing. Testing occurred in brightly lit (∼1000 lux) open field arenas covered in bedding. A normal food pellet was placed into the brightly lit center and the latency for an animal to approach and bite the pellet was recorded over a 12 min session. Immediately following the session, animals were placed into their home cage and allowed to eat a weighed food pellet to assess hunger drive. *Cookie test*: The cookie test apparatus contains three identically sized chambers (40 cm × 20 cm × 20 cm) separated by two offset dividers. The outer walls of each chamber were clear and the only difference between each was the color of the divider, with one divider shaded white and the other black. One week prior to experimentation, mice were habituated in their home cage to a piece (2 ± 1 g) of Keebler Fudge Stripe Cookie (Kellogg’s Company, Battle Creek, MI, USA). On the first and second day of testing, a piece of cookie (2 ± 1 g) was placed into the chamber separated by the black divider. Mice were then placed into the opposing chamber separated by the white divider and monitored for 10 min. The latency for each mouse to bite the cookie was recorded. Data from day two of the cookie test are presented, after mice acclimated to the anxiogenic environment and their behavior is driven more prominently by reward-seeking, resulting in a decrease in latency to bite (Isingrini et al., 2011; Soumier and Sibille, 2014).

### Pharmacokinetic Study of α5-PAM

Adult (8 weeks old) male and female C57BL/6J (Military Farm, Belgrade, Serbia) were divided into six groups corresponding to pre-determined time intervals following a single 30 mg/kg α5-PAM injection (5, 10, 20, 40, 60, and 180 min). At 30 mg/kg α5-PAM administration has high affinity for the α5-subunit and lower affinity for GABA_A_-receptors containing other subunits (Savic et al., 2010). As the goal of this study was to selectively evaluate positive modulation of α5-containing GABA_A_ receptors to stress-induced behaviors, we chose this dose to conduct preliminary pharmacokinetic analyses. Blood samples were collected in heparinized syringes via cardiac puncture of mice anesthetized with ketamine (100 mg/kg i.p.; 10% Ketamidor, Richter Pharma Ag, Wels, Austria), and centrifuged at 2500 rpm for 10 min to obtain plasma. Thereafter, mice were decapitated and brains were weighed, homogenized in 2 mL of methanol and centrifuged at 6000 rpm for 20 min. To determine the concentration of α5-PAM in plasma and supernatants of brain tissue homogenates, α5-PAM was extracted by solid phase extraction using Oasis HLB cartridges (Waters Corporation, Milford, MA, USA). The procedure of sample preparation and determination of α5-PAM by ultra performance liquid chromatography–tandem mass spectrometry (UPLC–MS/MS) with Thermo Scientific Accela 600 UPLC system connected to a Thermo Scientific TSQ Quantum Access MAX triple quadrupole mass spectrometer (Thermo Fisher Scientific, San Jose, CA, USA) equipped with electrospray ionization (ESI) source, is described in Obradovic et al. (2014).

### Plasma Protein and Brain Tissue Binding Studies

The rapid equilibrium dialysis assay used to determine free fraction of α5-PAM in mouse plasma and brain tissue was the same as in Obradovic et al. (2014).

### Tissue Samples and Micropunch Procedure

Twenty-four hours after the final behavioral test, mice were injected with either an acute dose of α5-PAM or vehicle 30 min prior to rapid perfusion (2 ml 4%PFA). Brains were rapidly dissected and flash frozen on dry ice. 160 μm thick coronal sections were obtained using a cryostat. The prelimbic cortex and dorsal hippocampus were dissected with a 1 mm diameter micropunch and tissue samples were then frozen at -80°C. Total RNA was extracted with an RNAeasy FFPE kit (Qiagen, Germantown, MD, USA) and tested for purity on a NanoDrop Spectrophotometer (260/280 ≥ 1.9). RNA concentration and integrity (RIN > 7) were then assessed using the Agilent RNA6000 Pico-Kit and Agilent 2100 Bioanalyzer (Agilent Technologies, Santa Clara, CA, USA).

### Real-Time Quantitative PCR

Approximately 90 ng of total RNA was used to generate cDNA via the qScript^TM^ cDNA Supermix synthesis kit (Quanta Biosciences, Gaithersburg, MD, USA). Real-time quantitative polymerase chain (qPCR) reactions were carried out using SYBR green fluorescence signal (Invitrogen, Carlsbad, CA, USA) and an Opticon Monitor DNA Engine (Bio-Rad, Berkeley, CA, USA). Gene of interest was the α5-subunit of the GABA_A_ receptor (*Gabra5*). Samples were run in triplicate and Δ*C*_t_ values were calculated by comparing the *C*_t_ values of *Gabra5* to the geometric mean of two reference genes, β-Actin and glyceraldehyde 3-phosphate dehydrogenase (*Gapdh*). Arbitrary signal intensity was calculated as 2^-ΔCt∗^10,000.

### Surface Biotinylation

Dissected hippocampi were processed as described in Pabba et al. (2014) with minor modifications.

### Antibodies

The following antibodies and their dilutions were used in this study: rabbit polyclonal anti-α5 (1:750; Thermo Fisher Scientific, Waltham, MA, USA), rabbit monoclonal anti-β_1_-N^+^/K^+^-ATPase (NK-ATPase) (1:1000; Abcam, Cambridge, MA, USA), and mouse monoclonal anti-β-actin (1:100; Abcam, Cambridge, MA, USA).

### Western Blotting

Western blotting was performed using 5 μg of protein resolved on 8% SDS-PAGE and transferred onto PVDF membranes and developed using IR-Dye conjugated secondary antibody and detection using LI-COR (NE, USA). Quantification of bands was performed using Image Studio^TM^ Software (LI-COR, NE, USA). We analyzed α5 surface biotinylation in vehicle versus drug treated mice (*N* = 6 females and 4 males per group). Total α5 band intensities were normalized to β-actin, whereas surface α5 band intensities were normalized to NK-ATPase.

### *Z*-Score Generation and Statistical Analysis

To assess the consistency of effects of stress and drug treatment on overall stress-induced behavior, we calculated behavioral *z*-scores using the formula below. These normalized scores integrate measures of behavioral dimensions (e.g., emotionality) over time and tests, yielding a combined measure of the UCMS-induced behavioral syndrome (Guilloux et al., 2011).

$$Z=\frac{X-\mu }{\sigma }$$

Briefly, for each behavioral measure a *Z*-score was calculated for an individual animal indicating how many standard deviations (σ) a given observation (*X*) is from the mean of the control group (μ). These scores were averaged within test such that each test had the same weight, and then averaged across all four tests (EPM, OFT, NSF, cookie test) to obtain a composite emotionality *Z*-score for each mouse. In short, random or opposite behavior across tests yield null *z*-scores, whereas significant and/or trend-like behaviors across tests and consistently in the same directions yield high *z*-scores.

Behavioral and gene expression data were analyzed with a two-way (sex × treatment) or one-way analysis of variance (ANOVA). The Holm–Sidak correction was used to determine group differences only following a significant main effect or interaction in the ANOVA model. In one specific experiment (see “α5-PAM Has No Anti-stress Properties in Male Mice Even after Augmenting UCMS with Single Housing and Increasing the Dose of α5-PAM”) *a priori* predictions led to planned comparison using unpaired two-tailed *t*-tests of UCMS-vehicle treated mice and non-stressed (NS-) vehicle treated mice.

## Results

### Systemic Administration of α5-PAM Leads to Rapid Increase in Brain Levels in Female and Male Mice

**FIGURE 1:**
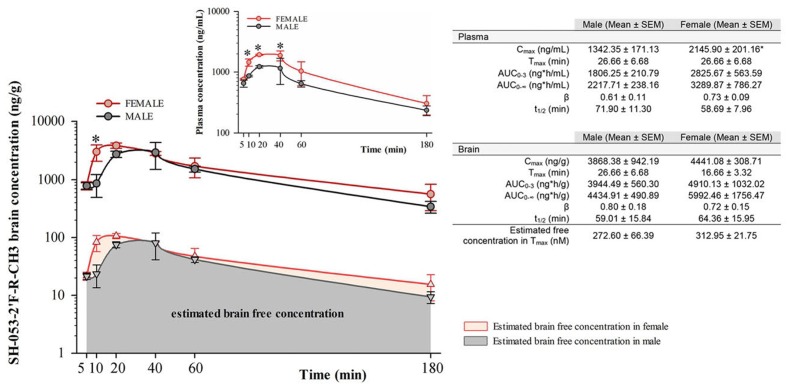
**Pharmacokinetic analysis of α5-PAM brain and plasma levels in female and male mice.** Brain (main figure) and plasma (inset) concentration–time profiles of α5-PAM (30 mg/kg) after intraperitoneal administration (*n* = 3 per time point), with estimated free brain concentration in female and male mice. ^∗^*p* < 0.05 between female and male mice in different time-points, according to Tukey *post hoc* test after two-way repeated ANOVA. The calculated plasma and brain pharmacokinetic parameters of α5-PAM are tabularly presented on the right. ^∗^*p* < 0.05 between female and male mice according to *t*-test.

Free concentrations of α5-PAM were calculated by multiplying the total plasma and brain concentrations with the appropriate free fractions (7.44% for plasma and 2.73% for brain tissue) determined by rapid equilibrium dialysis. Two-way repeated measure ANOVA for total brain concentration revealed a statistically significant difference between female and male mice [*F*(1,4) = 7.83, *p* < 0.05], and between time points [*F*(5,20) = 6.89, *p* < 0.001] with no significant interaction [*F*(5,20) = 1.05, *p* > 0.05]. α5-PAM plasma concentration were higher in female than male mice [*F*(1,4) = 16.54; *p* < 0.05] with a significant effect of time [*F*(5,20) = 12.27, *p* < 0.001] and no interaction [*F*(5,20) = 1.02, *p* > 0.05]. *Post hoc* comparisons revealed significant differences between female and male mice in plasma *C*_max_ [*t*(4) = -3.04, *p* < 0.05, **Figure 1**], confirming that higher concentrations were obtained in female mice. Furthermore, the AUC_0-3_ in brain is approximately 1.25X higher for females (4910.13 ± 1032.02) than males (3944.49 ± 560.30), suggesting that a cumulative 25% more ligand is available in the brain of female mice, after the same dose. However, at 30 min, the amount of ligand in the brain of male and female mice was not statistically different (**Figure 1**). For this reason, we initially chose the 30 mg/kg dose and 30 min time point for evaluating the acute behavioral effects of α5-PAM in male and female mice. In behavioral experiments, acute treated male and female mice received a 30 mg/kg injection of α5-PAM with 48 h in between, when no existing compound remained in plasma or brain in either male or females based on *t*_1/2_ (**Figure 1**). Thus, we consider this an acute treatment prior to each behavioral test (See Discussion).

### α5-PAM Reduces the Stress-Induced Behaviors in Female, but Not Male Mice

**FIGURE 2:**
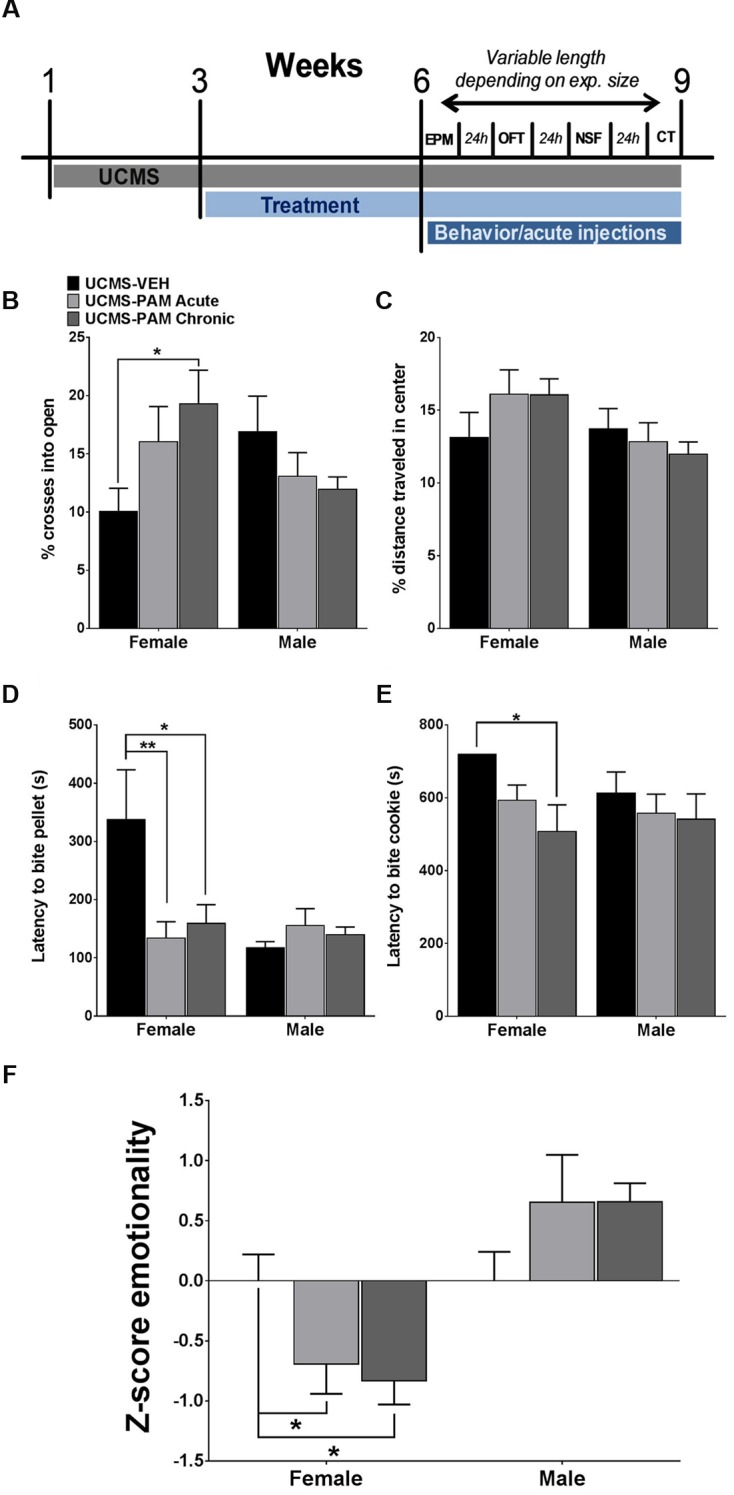
**Effect of α5-PAM on stress behavior induced by UCMS in female and male mice. (A)** Timeline of experimental procedures. Tick marks indicate the beginning of a week. Depending on number of animals, behavioral testing took between 1 and 3 weeks. **(B)** Female mice treated with α5-PAM chronically had increased percentage of open arm entries relative to UCMS-VEH mice, with no effect of treatment in male mice. **(C)** No effect of either acute or chronic α5-PAM treatment in the OFT of either male or female mice after UCMS. **(D)** Treatment with acute and chronic α5-PAM reduced the latency to bite a food pellet in the NSF in female but not male mice. **(E)** Chronic α5-PAM treatment reduced the latency to bite a piece of cookie in the cookie test in female mice, while a trend in the same direction was observed following acute treatment. No significant effect of α5-PAM was detected in males. **(F)** Significant anti-stress effect of both acute and chronic α5-PAM treatment on female, but not male, *Z*-emotionality scores, Data represent mean ± SEM (*N* = 8–10 per group). ^∗^*p* < 0.05, ^∗∗^*p* < 0.01.

We first sought to examine whether acute or chronic α5-PAM treatment would have an anti-stress effect in female and male mice exposed to UCMS. Due to the large number of mice (total *N* = 54) and necessity for randomness of stressors in order to produce a robust UCMS effect (Guilloux et al., 2011), as well as the reported lack of baseline (non-stressed) effects of α5-PAM (Savic et al., 2008, 2010), we did not include non-stressed mice in this initial experiment (see “α5-PAM Reduces the Stress-Induced Behavioral Emotionality of Female Mice to Non-stressed Control Levels” and “α5-PAM Has No Anti-stress Properties in Male Mice Even after Augmenting UCMS with Single Housing and Increasing the Dose of α5-PAM” for non-stressed comparisons). **Figure 2A** illustrates a timeline of all experimental procedures. In the EPM, we found a significant interaction between sex and treatment [*F*(2,48) = 4.094, *p* < 0.05], with *post hoc* analysis revealing that chronic α5-PAM treatment exhibiting an anti-stress effect on the percentage of entries into open arms (**Figure 2B**). No significant differences were observed in male mice following stress (*p* > 0.05). In the OFT, we did not detect any significant main effects (**Figure 2C**; *p* > 0.05) or an interaction between effects (*p* > 0.05). While EPM and OFT measures are corrected for changes in locomotion, it is important to note that no main effect of treatment, sex, or interaction on locomotion in the OFT was observed (Supplementary Figure S1C; *p* > 0.05).

**FIGURE 3:**
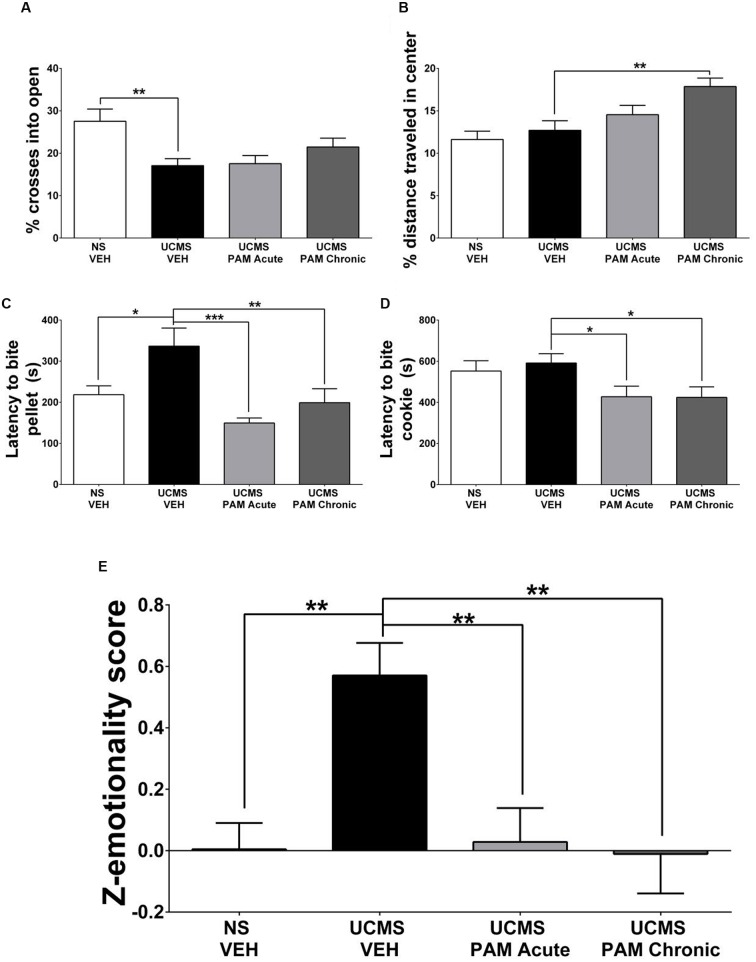
**Treatment with α5-PAM normalizes stress behavior to non-stressed levels in female mice. (A)** UCMS reduces the percentage of entries into the open arms of the EPM. **(B)** Chronic treatment with α5-PAM increases the percentage of total distance traveled in the center of the open field. **(C)** UCMS increases the latency to bite in the NSF compared to NS-VEH treated animals, while both acute and chronic α5-PAM treatment reduces the increased latency following UCMS. **(D)** Acute and chronic α5-PAM treatment reduces the latency to bite a piece of cookie in the cookie test compared to UCMS-VEH treated mice. **(E)** Overall emotionality *Z*-score indicates an increase in emotionality in the UCMS-VEH treated mice that is normalized following acute and chronic α5-PAM administration. Data represent mean ± SEM (*N* = 15–20 per group). ^∗^*p* < 0.05, ^∗∗^*p* < 0.01, ^∗∗∗^*p* < 0.001.

We next examined the anti-stress properties of α5-PAM in the NSF, typically robustly affected by UCMS (Lin and Sibille, 2015), and cookie test, which taps into the anhedonic dimension of stress-induced symptoms. In the NSF, we again observed a significant interaction between sex and treatment [*F*(2,48) = 3.930, *p* < 0.05] and a similar pattern of behavior in female mice, with acute (*p* < 0.01) and chronic (*p* < 0.05) α5-PAM treatment reducing latency to bite compared to UCMS-vehicle treated animals. Again, no effect of either acute or chronic α5-PAM was observed in male mice (**Figure 2D**; *p* > 0.05). Importantly, neither sex [*F*(1,48) = 1.344, *p* > 0.05] nor α5-PAM treatment [*F*(2,48) = 1.721, *p* > 0.05] altered the percentage of weight lost after 24 h of food deprivation prior to the NSF test with no interaction between the two factors [Supplementary Figure S1A; *F*(2,48) = 1.775, *p* > 0.05]. Likewise, there was no effect of treatment on the amount of normal chow consumed in the home cage 8 min after the NSF [Supplementary Figure S1B; *F*(2,48) = 1.441, *p* > 0.05], though males did consume more relative to females [*F*(1,48) = 4.061, *p* < 0.05]. Together, this suggests that differences in latency to bite are not due to a non-specific effect of treatment on hunger. In the cookie test, two-way ANOVA indicated a significant main effect of treatment [*F*(2,48) = 3.504, *p* < 0.05]. Follow-up comparisons uncovered a significant decrease in latency to bite the cookie in female mice following chronic α5-PAM administration (*p* < 0.05). No significant difference in latency to bite was observed in male mice after acute or chronic α5-PAM treatment (**Figure 3E**). Given significant overlap between anxiety and depression behavior in both humans and rodents, we examined the combined anti-stress effect of α5-PAM on emotionality behavior via *Z*-normalization across tests (**Figure 2F**). We found a significant main effect of sex [*F*(1,48) = 19.66, *p* < 0.05] and an interaction between sex and treatment [*F*(2,48) = 4.927, *p* < 0.05]. Subsequent multiple comparisons revealed an anti-stress effect of chronic (*p* < 0.05) and acute α5-PAM (*p* < 0.05), but no effect in male mice (**Figure 2E**; *p* > 0.05). Together, these data indicate that acute and chronic treatments with α5-PAM result in a rapid reduction of stress-induced behaviors in female but not in male mice.

### α5-PAM Reduces the Stress-Induced Behavioral Emotionality of Female Mice to Non-Stressed Control Levels

Our first exploratory experiment suggested that treatment with α5-PAM has anti-stress activity in UCMS-exposed female mice. Therefore, we sought to replicate these findings in a separate cohort of female mice. Specifically, we asked whether acute or chronic treatment with α5-PAM could reverse UCMS-induced emotionality behaviors back to levels measured in non-stressed control mice. In the EPM a significant effect of treatment was observed in the percentage of crosses an animal made into the open arms [*F*(3,66) = 5.069, *p* < 0.01]. *Post hoc* comparisons indicated that UCMS-vehicle treated animals spent a smaller percentage of time in the open arms compared to non-stressed vehicle animals (**Figure 3B**; *p* < 0.001), although no effect of either acute or chronic α5-PAM treatment was observed (*p* > 0.05). In the OFT, mice treated chronically with α5-PAM had a higher percentage of their total distance traveled (**Figure 3B**; *p* < 0.01) in the center of the arena compared to vehicle-treated UCMS-exposed animals.

In the NSF test, a significant effect of treatment was observed in the latency to bite the food pellet [*F*(3,66) = 7.261, *p* < 0.001]. *Post hoc* comparisons indicated that UCMS-vehicle treated animals took longer to bite the food pellet than non-stressed vehicle animals (*p* < 0.05), and that both acute (*p* < 0.001) and chronic (*p* < 0.01) α5-PAM treatments reduced the latency to bite compared to UCMS-vehicle mice (**Figure 3C**). In the cookie test, there was a significant effect of treatment [*F*(3,66) = 3.065, *p* < 0.05] with *post hoc* comparisons revealing both acute (*p* < 0.05) and chronic (*p* < 0.05) α5-PAM treatment significantly reduced the latency to bite the cookie (**Figure 3D**).

Although the effect of stress was not consistent across all tests, we observed a convergent effect of UCMS on *Z*-emotionality scores [*F*(3,66) = 7.125, *p* < 0.001], with *post hoc* comparisons highlighting an increase in behavioral emotionality of UCMS-vehicle treated animals compared to non-stressed vehicle animals (**Figure 3E**; *p* < 0.001). Acute (*p* < 0.001) and chronic (*p* < 0.001) α5-PAM treatment significantly decreased stress-induced *Z*-emotionality scores compared to the UCMS-vehicle group. The acute and chronic α5-PAM treatment groups were not statistically different from the non-stressed vehicle group (**Figure 3E**; *p* > 0.05). Together these findings replicated and confirmed the prior results suggesting an anti-stress effect of α5-PAM in female mice (**Figure 2**) and highlight the value of *z*-scoring at identifying consistent and convergent behavioral changes within mouse across multiple tests.

### α5-PAM Has No Anti-Stress Properties in Male Mice Even After Augmenting UCMS with Single Housing and Increasing the Dose of α5-PAM

**FIGURE 4:**
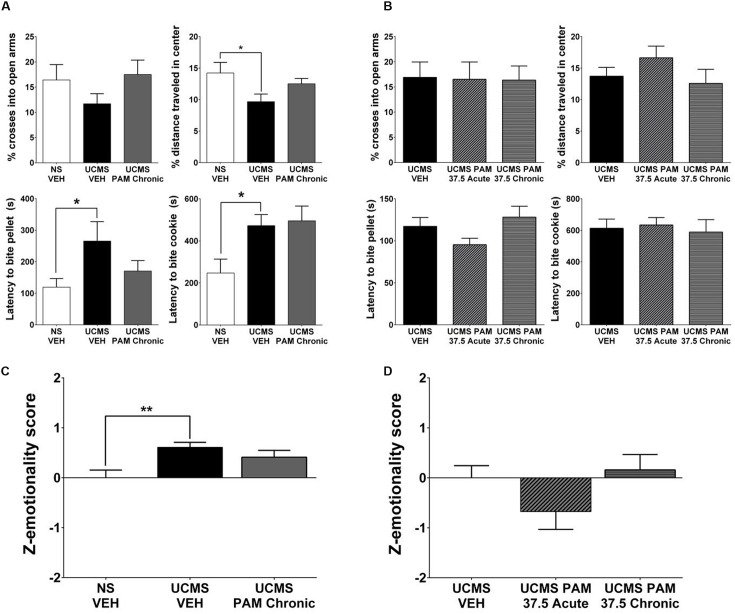
**α5-PAM has no anti-stress properties in male mice even after augmenting UCMS with single housing and increased α5-PAM dose. (A)** UCMS combined with isolation housing results in no change in EPM behavior, but a significant decrease in the percentage of distance traveled in the OF, an increase in the latency to bite the pellet in the NSF, and an increase in latency to bite the cookie in the cookie test. No significant effect in any individual test of chronic α5-PAM treatment. **(B)** No change in stress induced behaviors in the EPM, OFT, NSF, or cookie test following acute or chronic treatment with 37.5 mg/kg α5-PAM. **(C)** Overall emotionality *Z*-scores are increased following UCMS-VEH treatment compared to NS-VEH mice. α5-PAM treatment does not remediate the increased emotionality induced by UCMS in male mice. **(D)** No effect of acute or chronic α5-PAM on stress-induced behaviors in male mice. Data represent mean ± SEM (*N* = 12 per group). ^∗^*p* < 0.05, ^∗∗^*p* < 0.01.

Similarly as for the effect in female mice, we sought to replicate the lack of α5-PAM effect observed in our preliminary study in an independent cohort. Moreover, given the inherent variability of the UCMS paradigm and the fact that the phenotype can be more robust in female compared to male mice (Guilloux et al., 2011), we conducted an experiment in which we single-housed male mice for the final 2 weeks of UCMS and throughout behavioral testing to potentiate UCMS-induced phenotypes, as previously described in Surget et al. (2009) and Ma et al. (2011). As shown in **Figure 4A**, planned comparisons indicate that the combination of UCMS and social isolation did not affect behavior in the EPM, but resulted in a decrease in the percent distance traveled in the center of the OF [*t*(22) = 2.184, *p* < 0.05], increased latency to bite a food pellet in the NSF [*t*(22) = 2.088, *p* < 0.05], and increased latency to bite in the cookie test [*t*(22) = 2.075, *p* < 0.05] relative to non-stressed vehicle-treated animals. Consistent with our prior observations, chronic treatment with α5-PAM did not alter the UCMS-induced behavioral phenotype of male mice in either the EPM, OFT, NSF or cookie test (**Figure 4A**; *p* > 0.05). Summary emotionality *Z*-scores indicated that combined UCMS and isolation housing robustly increases emotionality behavior in male mice compared to non-stressed controls [**Figure 4D**; *t*(22) = 3.331, *p* < 0.01], and confirmed the lack of effect of chronic α5-PAM treatment in male mice [*p* > 0.05].

Additionally, although not statistically significant at 30 min, pharmacokinetic data indicate elevated accumulation of α5-PAM in the brain of female compared to male mice during the first 30 min post injection (**Figure 1**). Therefore we tested a 25% higher dose (37.5 mg/kg) in males. We found it also failed to reduce stress-induced behavior across all individual measures in the EPM, OFT, NSF, and cookie test (**Figure 4B**; *p* > 0.05) in male mice. Composite emotionality *Z*-scores confirm, similar to the 30 mg/kg dose (**Figure 2E**), a lack of anti-stress activity of 37.5 mg/kg α5-PAM (**Figure 4D**; *p >* 0.05).

### α5-PAM Treatment Increases Gabra5 Transcript Levels in the Prelimbic Cortex and Hippocampus of Female Mice and Levels Are Correlated with Stress Behavior Only in Female Mice

**FIGURE 5:**
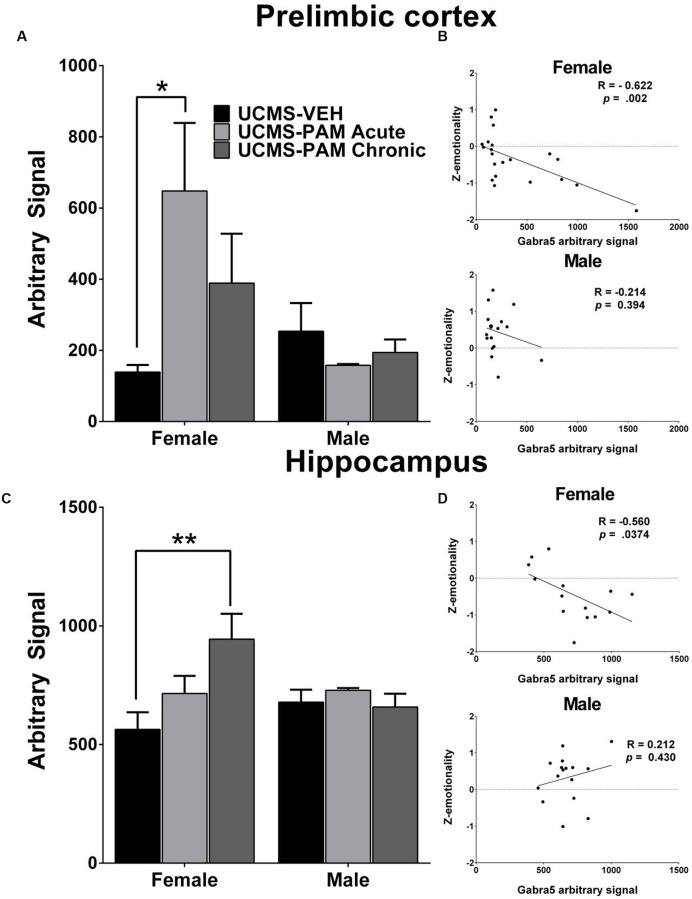
***Gabra5* transcript expression correlates with stress behavior and is increased after α5-PAM administration in the prelimbic cortex and hippocampus of female, but not male mice. (A)** Acute treatment with α5-PAM increases *Gabra5* expression in the prelimbic cortex of female mice exposed to UCMS. No change in *Gabra5* expression was observed following treatment in male mice. **(B)** *Z*-emotionality scores significantly correlate with prelimbic cortex *Gabra5* expression in female (top), but not male (bottom) mice **(C)** Chronic treatment with α5-PAM increases *Gabra5* expression in the hippocampus of female mice exposed to UCMS and compared to animals treated with α5-PAM acutely. No change in *Gabra5* expression was observed following either acute or chronic treatment in male mice. **(D)** *Z*-emotionality scores significantly correlate with hippocampal *Gabra5* expression in female (top), but not male (bottom) mice. Data represent mean ± SEM (*N* = 8–10 per group). ^∗^*p* < 0.05, ^∗∗^*p* < 0.01.

**FIGURE 6:**
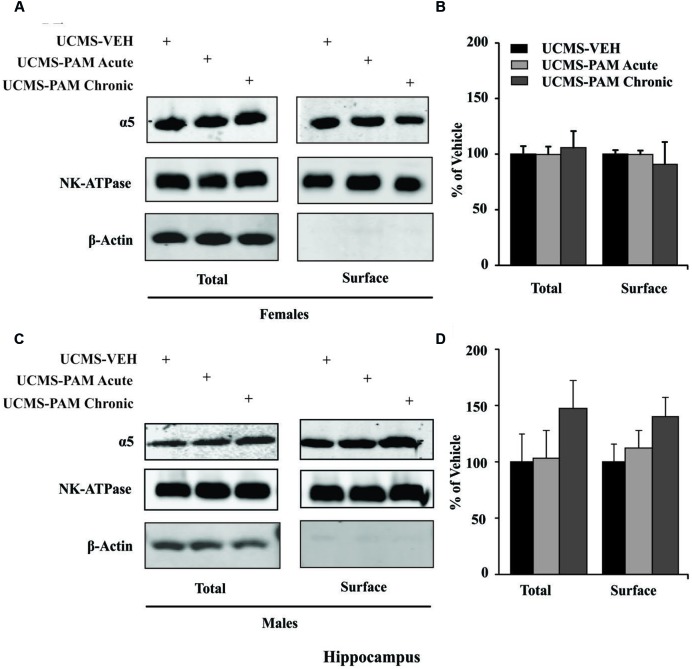
**No change in either α5 total or surface protein level in the hippocampus of male and female mice. (A,C)** Representative blots of two housekeeping proteins (NK-ATPase, β-Actin) as well as α5 at the surface and total protein levels following UCMS and either vehicle, acute, or chronic α5-PAM treatment in female **(A)** or male **(C)** mice. **(B,D)**. No significant effect of treatment observed on either total or surface α5 protein in female **(B)** or male **(D)** mice.

To evaluate the possibility that changes in α5-subunit expression might explain the sex-specific behavioral effects of treatment, we assessed levels of α5 gene (*Gabra5*) and protein expression within the prelimbic cortex and hippocampus of treated and untreated UCMS-exposed mice. Results show no difference in expression of the α5-subunit in either the prelimbic cortex or hippocampus as a factor of sex following UCMS exposure (**Figures 5A,C**, black bars; *p* > 0.05). In prelimbic cortex, we observed a significant interaction between sex and treatment [*F*(2,32) = 3.508, *p* < 0.05], with *post hoc* comparisons indicating that acute α5-PAM treatment significantly increased α5 transcript expression in female mice (**Figure 5A**; *p* < 0.05). No effect of chronic treatment was observed in male or female mice (*p* > 0.05). In hippocampus, we again observed an interaction between sex and treatment [*F*(2,26) = 4.45, *p* < 0.05], with *post hoc* comparisons highlighting an increase in α5-subunit expression in females after chronic α5-PAM treatment (**Figure 5C**; *p* < 0.01). No changes in *Gabra5* gene expression were observed in males after either acute or chronic α5-PAM treatment (*p* > 0.05). Further supporting a link between *Gabra5* and stress-induced behavior are significant negative correlations between expression in the female prelimbic cortex (**Figure 5B**; *R* = -0.622, *p* < 0.01) and hippocampus (**Figure 5D**; *R* = -0.560, *p* < 0.05) and *Z*-emotionality scores. No such significant correlations were observed in male mice in either the prelimbic cortex (*R* = -0.214, *p* > 0.05) or hippocampus (*R* = 0.212, *p* > 0.05). We next sought to determine whether changes in RNA expression corresponded to α5 protein levels. We focused on the hippocampus due to high α5 levels in that region. Surprisingly, we detected no difference in either total or surface α5 protein expression following either acute or chronic α5-PAM treatment in female (**Figures 6A,B**; *p* > 0.05) or male mice (**Figures 6C,D**; *p* > 0.05).

## Discussion

### Treatment with α5-PAM Reduces Stress-Induced Behavior in Female, but Not Male Mice

We demonstrate that treatment with a GABA_A_ receptor PAM with high affinity for α5 subunit-containing subtype (α5-PAM) (Savic et al., 2010) consistently results in a reduction in stress-induced behaviors in female mice exposed to UCMS. This effect was replicated across two independent cohorts (**Figures 2** and **3**). In contrast, treatment with α5-PAM did not alter stress-induced behaviors in male mice (**Figure 2**), even after enhancing the UCMS effect in a second cohort with isolation housing (**Figures 4A,C**). The observation that acute and chronic α5-PAM treatments were effective in blocking UCMS-induced behavioral emotionality in female mice (**Figures 2** and **3**) raises the possibility that targeting the α5-containing GABA_A_ receptors could have rapid anti-stress effects.

Pharmacokinetic studies (**Figure 1**) suggest that the sex-specific behavioral differences are not likely to be explained by sex-dependent drug availability. Females displayed time-dependent elevated α5-PAM plasma levels compared to males (**Figure 1**). However, no effect of α5-PAM was observed in males at times when plasma and total brain levels were similar to females (30 min; **Figure 1**) or when a 25% higher dose of α5-PAM was used (**Figure 4**). Further,no differences in the free concentration of brain SH-053-2′F-R-CH3 were observed at any time point. Together, these data indicate that it is unlikely our sex-specific behavioral differences were solely due to pharmacokinetic differences. Note that, even at *T*_max_, the estimated free brain concentrations in males (273nM) and females (313nM) were several times lower than the reported *K*_i_ values of α5-PAM at non-α5-GABA_A_ receptors (Savic et al., 2010), thus demonstrating selectivity of modulatory action of the ligand on mouse behavior in the settings used. Despite slight differences in the effect of acute versus chronic treatment (**Figures 3A–D**, Supplementary Figure S1), the data were qualitatively consistent across cohorts of mice in both sexes (**Figures 2–4**).

### Sex-Dependent Effects of α5-PAM Treatment Parallel Human Sex-Dependent Molecular Deficits and Highlight Modulation of Extrasynaptic GABA_A_ as a Potential Novel Therapeutic Modality

The observed sex difference in preclinical testing is potentially clinically interesting since women are twice as likely than men to experience a single episode of depression (Kessler, 2003) and display higher morbidity risk (Piccinelli and Wilkinson, 2000). These differences are not explained by diagnostic criteria, suggesting a biological predisposition in women (Angst and Dobler-Mikola, 1984). Interestingly, evidence suggest that dysfunction in SST interneurons may be more robust in females. In two frontocortical regions critical for emotion regulation, the sgACC and dlPFC, decreases in markers of dendritic targeting interneurons were more pronounced in female MDD compared to male MDD subjects and their matched controls (Sibille et al., 2010; Tripp et al., 2011, 2012; Seney et al., 2013). This decrease in SST expression spanned all cortical layers in the sgACC of MDD subjects with greater effect sizes and significance in female subjects, suggesting a cellular vulnerability affecting all cortical SST neurons in MDD and more robustly in females (Seney et al., 2014).

In Lin and Sibille (2015), we showed that UCMS specifically affects expression of SST and GABA synthesizing molecules in SST neurons. The current pharmacological and behavioral results indicate that compounds that enhance α5-GABA_A_R mediated inhibition postsynaptic to SST cells may overcome or bypass the impact of the intrinsic vulnerability of SST cells to stress. The fact that the potential therapeutic action of α5-PAM involves these cortical deficits is causally supported by our finding that acute treatment with α5-PAM upregulates expression of the *Gabra5* gene exclusively in female mice within the prelimbic cortex (homologous to the human sgACC), and that levels of the *Gabra5* gene are correlated with lower stress-induced emotionality behavior. In prelimbic cortex, low expression of total α5 protein precluded our ability to compare transcript and protein levels. In the hippocampus, where α5 mRNA and protein are highly expressed, we observed an increase in transcript expression following chronic α5-PAM treatment in female mice and, as in the cortex, a negative correlation between *Gabra5* transcript levels and emotionality behavior, suggesting an important role for *Gabra5* in stress-induced behaviors (**Figures 5C,D**). However, no corresponding change in either total or surface protein level was observed (**Figure 6**). Contributing to this discrepancy may be the fivefold to sixfold increase in protein half-life relative to mRNA, which results in greater dynamic mRNA regulation (Schwanhausser et al., 2011), or simply that at the time of sacrifice, translation of the α5 transcript was not elevated. It is worth noting that chronic α5-PAM treatment in female mice produced anti-stress effects with no down-regulation of the *Gabra5* transcript, commonly observed following chronic benzodiazepine administration (Uusi-Oukari and Korpi, 2010), which potentially explains the development of drug tolerance to benzodiazepine-like pan-GABA_A_ receptor modulators (Vinkers and Olivier, 2012). In fact, sustained upregulation of α5 protein has been reported after exposure to anesthetics that act primarily as GABA_A_ receptor modulators (Zurek et al., 2014). These findings further highlight the specificity of α5-PAM for α5-subunit containing GABA_A_ receptors and alleviate concerns regarding a decrease in efficacy following chronic treatment.

Converging evidence from clinical data suggests an involvement of extrasynaptic GABA_A_ receptors in psychiatric illness. Linkage studies have identified a susceptibility locus within the *GABRA5* gene (15q11-q13) for bipolar depression (Otani et al., 2005; Kato, 2007). Post-mortem studies have further examined the link between *GABRA5* gene expression and depression, with mixed findings. In the ACC and dlPFC, data suggest that expression of the *GABRA5* gene is increased in cortical layers 2–6 of subjects with bipolar depression and those with MDD (Choudary et al., 2005). In post-mortem dlPFC tissue from depressed suicides, no difference in *GABRA5* expression was observed (Merali et al., 2004). Similarly, in the amygdala of female post-mortem subjects, where decreases in SST and other GABA interneuron markers are present, no change in *GABRA5* gene expression was observed between MDD and matched controls (Guilloux et al., 2012). While this is the first study to demonstrate anti-stress effects of a selective α5-PAM compound, other studies have found that compounds with mixed α5 and α2 specificity do have anxiolytic effects in rodents (Savic et al., 2008, 2010). Moreover a recent study in the amygdala suggests that generalization of fear and anxiety is mediated by α5-subunit containing neurons in the central nucleus of the amygdala (Botta et al., 2015). Consistent with the role of the α5-subunit in anxiety, the anxiolytic effect of diazepam was manifest in mice rendered insensitive to its modulatory action at all GABA_A_R subunits other than α5-GABA_A_Rs (Behlke et al., 2016). Taken together, the data support a potential role for the α5-GABA_A_ receptor in stress-associated disorders, including anxiety and depression, though it is an area to study further, especially as it pertains to potential sex differences.

Data characterizing the expression of α5-GABA_A_ receptors in stress models are sparse, and our report is the first to examine potential sex-specific effects. Previous reports suggest that expression of the α5-subunit is increased in the frontal cortex following prolonged social isolation in male mice (Matsumoto et al., 2007). Expression of another extrasynaptic GABA_A_ receptor subunit, the δ-subunit, is also increased after social isolation, a finding that correlates with increased tonic inhibition (Serra et al., 2006). A notable difference, however, is that the δ-subunit is not restricted to the dendritic compartment, as is the case for the α5-containing GABA_A_ receptors (see Introduction). Interestingly, expression of extrasynaptic δ-GABA_A_ receptor is modulated across the estrous cycle and sensitive to the neurosteroid progesterone, an effect that is rapid and does not require activation of neurosteroid receptors (Maguire et al., 2005; Maguire and Mody, 2007). Expression of extrasynaptic GABA_A_ receptors is decreased at parturition and increased postpartum, providing support for hormonal modulation of these extrasynaptic receptors. Further, estrogen treatment itself has long been known to upregulate GABA_A_ receptors in the brain of rodents (Maggi and Perez, 1986), though a complete evaluation of the exact subunit combinations altered has not been conducted. Given our observed sex specific effects of treatment with α5-PAM, it is reasonable to hypothesize that interaction with circulating steroid hormones (e.g., estrogens) and extrasynaptic GABA_A_ receptors (e.g., containing the α5 and δ-subunits), may be influencing this finding. Further supporting this notion is our finding that acute and chronic treatments selectively upregulate α5-GABA_A_ receptor expression in the prelimbic cortex and hippocampus (**Figure 5**). Together, these preclinical data support the involvement of extrasynaptic GABA_A_ receptors in the stress response and indicate sex-specific regulation of receptor expression.

### Limitations

The present study, while potentially suggesting a novel sex-dependent therapeutic modality for antidepressant treatment, is not without limitations. The authors acknowledge the inherent variability of the UCMS procedure, where the phenotype typically ranges in severity and may not be observed systematically and statistically in every single behavioral test, when analyzed in isolation (Guilloux et al., 2011). This variability mirrors what is observed in MDD, where diagnosis is not based on a single observable behavior, but rather a convergent set of symptoms from multiple measures across multiple time points (e.g., physiological/somatic symptoms and affective symptoms). For this reason we have adopted a *Z*-normalization procedure to assess the consistency of behavior across tests and to increase the translational value of the UCMS model (Surget et al., 2009; Edgar et al., 2011; Guilloux et al., 2011; Quesseveur et al., 2015). Harnessing this approach, we demonstrate that in multiple cohorts, despite modest effect size and minor differences in significance across specific tests, α5-PAM treatment in female animals consistently normalized stress behaviors (**Figures 2** and **3**) while male animals were unaffected (**Figures 2** and **4**). An additional limitation is the use of multiple injections separated by 48 h for our acute α5-PAM treated mice. While pharmacokinetic data indicate no drug should remain in either the plasma or brain 48 h after a single injection (**Figure 1**), we cannot rule out the possibility that a single injection resulted in a lasting neuroplastic change that could have been compounded by subsequent injections. Regardless, this factor did not impact our ability to detect sex-dependent behavioral differences in stress behavior (**Figures 2–4**).

### Therapeutic Potential of Targeting α5-Containing GABA_A_ Receptors

Here, we demonstrate that a GABA_A_ α5-selective PAM can reduce stress behavior induced by UCMS to non-stressed levels when administered acutely and chronically to female mice (**Figures 2** and **3**). The effect on anxiety-like behaviors in females was mild, although behavioral *Z*-score normalization identified consistent behavioral responses across tests, and it was replicated across two independent cohorts (**Figures 2** and **3**). By contrast, we observed a consistent lack of effect in male mice across separate cohorts, regardless of treatment regimen or dose (**Figures 4C,D**). This lack of effect in males was not explained by pharmacokinetic differences (**Figure 1**). These data confirm previous reports indicating a lack of anxiogenic/anxiolytic properties of specific α5-subunit modulation in male rodents either pharmacologically (Savic et al., 2008, 2010) or genetically (Collinson et al., 2002), although a recent restriction of GABA_A_-subtype function experiment in male mice suggests α5-mediated anxiolysis (Behlke et al., 2016). Interestingly, a recent study in male rats found that two negative allosteric modulators (NAM) selective for the α5-subunit could reverse stress-induced anhedonia and deficits in AMPAR-mediated synaptic transmission within the hippocampus (Fischell et al., 2015). While these findings appear at odds with the data presented here, with preclinical studies suggesting anxiogenic effects of α5-NAMs (Navarro et al., 2002), and with the clinical and human post-mortem literature, they are in accordance with the actions of the rapid-acting antidepressant ketamine (Berman et al., 2000; Li et al., 2010; Autry et al., 2011). Together, these studies suggest that basal GABA tone or baseline cellular activity, for which sex may be a mediating factor, may influence which type of α5-modulator (positive or negative) may produce a therapeutic benefit. Support for this contention comes from the finding that while α5 GABA_A_ receptor NAMs enhance memory in young rats, positive modulation is necessary to improve memory in older rats with excessive hippocampal activity and decreases in α5 transcript expression (Haberman et al., 2011; Koh et al., 2013). We believe that the same principle may exist in the UCMS condition, where animals are in a low GABA state due to dysfunction of SST cells (Lin and Sibille, 2015), and potentially in MDD where low GABA content and dendritic targeting interneuron dysfunction is observed in analogous brain regions.

## Conclusion

In summary, the present study supports the hypothesis that enhancing signaling specifically at α5-GABA_A_ receptors may represent a novel therapeutic approach for mood disorders, including anxiety and depression. Interestingly, this treatment approach may be more beneficial to women, who are more severely affected by MDD. Future experiments should further explore the sex-dependent effects of treatment and propose the exact mechanism by which modulation of α5-GABA_A_ receptors is exerting its effects in the UCMS condition.

## Author Contributions

SP designed and carried out behavioral and gene expression experiments, conducted data analysis, interpreted the data, and wrote the manuscript. BF, HO, and MLS helped design and carry out the behavioral experiments and provided manuscript feedback. MMP and JC synthesized and provided α5-PAM compound used in behavioral experiments and provided manuscript feedback. TT, BM, and MMS provided input on experimental design, conducted pharmacokinetic analysis, and provided manuscript methods and feedback. MP conducted protein analysis and provided manuscript feedback. BO and ES contributed to experimental design, interpretation of data, and input to the manuscript.

## Conflict of Interest Statement

ES, JC, and MMSare co-inventors on a recently filed US provisional patent application that covers compounds similar in the mechanism of action to SH-053. All the other authors declare that the research was conducted in the absence of any commercial or financial relationships that could be construed as a potential conflict of interest.
