# Supplementary material for: Sex-Dependent Anti-Stress Effect of an α5 Subunit Containing GABAA Receptor Positive Allosteric Modulator
Source: Front Pharmacol. 2016 Nov 22;7:446. doi: 10.3389/fphar.2016.00446 (PMC5118774; doi:10.3389/fphar.2016.00446)
Supplement: Supplementary file 1 [file Data_Sheet_1.doc]

**Supplemental Information for**

**Sex-dependent anti-stress effect of an α5 subunit containing GABAA receptor positive allosteric modulator**

Sean C Piantadosi^1,2^, Beverly French^2^, Michael M Poe^3^, Tamara Timić^4^, Bojan Marković^5^, Mohan Pabba^6^, Marianne L Seney^2^, Hyunjung Oh^1,2,7^, Beverley A. Orser^6^, Miroslav M Savić^4^, James M Cook^3^, Etienne Sibille^1,2,7,8^*

1. Figure S1
2. Table S1

**Figure S1**

**Figure S1. No effect of α5-PAM on control measures in the NSF and OFT**

**(A)** No effect of sex or treatment in the percentage of weight lost following food deprivation prior to the NSF **(B)** During the post-NSF food consumption test, no effect of treatment was observed, although males ate significantly more than females. **(C)** No effect of sex or treatment in total distance traveled within the OFT.

**Table S1. UCMS Schedule**

| **June** | (1) 17 | (2) 18 | (3) 19 | (4) 20 | (5) 21 | (6) 22 | (7) 23 |
| --- | --- | --- | --- | --- | --- | --- | --- |
| **Week1** | **Monday** | **Tuesday** | **Wednesday** | **Thursday** | **Friday** | **Saturday** | **Sunday** |
| **1** | **Transfer to 171 in afternoon** |  |  |  |  | no bedding overnight |  |
| **2** |  |  |  |  |  |  |  |
| **3** |  |  |  |  |  |  |  |
| **4** |  |  |  |  |  |  |  |
| **5** |  |  |  |  |  |  |  |
| **6** |  |  |  |  |  |  |  |
| **7** |  |  |  |  |  |  |  |
| **8** |  |  |  |  |  |  |  |
| **9** |  | cage tilt |  |  |  |  |  |
| **10** |  |  |  |  |  |  | Social stress: group cage exchange or two mice rotation |
| **11** |  |  |  |  | Forced Bath in big cage (15 minutes) |  |  |
| **12** |  |  |  | lights off (1.5 hours) |  |  |  |
| **1** |  |  | Bob cat urine 15 min |  |  |  |  |
| **2** |  |  |  |  |  |  |  |
| **3** |  |  |  |  |  |  |  |
| **4** |  |  |  |  |  |  |  |
| **5** | house and nestlets removed |  |  |  |  |  |  |
| **6** |  |  |  |  | no bedding overnight |  |  |
| **7** |  |  |  |  |  |  |  |
| **8** |  |  |  |  |  |  |  |
| **9** |  |  |  |  |  |  |  |
| **10** |  |  |  |  |  |  |  |
| **11** |  |  |  |  |  |  |  |
| **12** |  |  |  |  |  |  |  |

| **June** | 24 | 25 | 26 | 27 | 28 | 29 | 30 |
| --- | --- | --- | --- | --- | --- | --- | --- |
| **Week2** | **Monday** | **Tuesday** | **Wednesday** | **Thursday** | **Friday** | **Saturday** | **Sunday** |
| **1** |  |  |  | wet bedding |  | reduced space (divider) + cage change |  |
| **2** |  |  |  |  |  |  |  |
| **3** |  |  |  |  |  |  |  |
| **4** |  |  |  |  |  |  |  |
| **5** |  |  |  |  |  |  |  |
| **6** |  |  |  |  |  |  |  |
| **7** |  | cage tilt (2 hours) |  |  |  |  |  |
| **8** |  |  |  |  |  |  |  |
| **9** |  |  |  | restraint (20 min) | reduced space (divider) + cage change |  |  |
| **10** |  |  |  |  |  |  |  |
| **11** |  |  |  | new bedding |  |  |  |
| **12** |  |  |  |  |  |  |  |
| **1** |  |  |  |  |  |  |  |
| **2** |  |  |  |  |  |  | forced bath in big cage (30 min) |
| **3** |  |  |  |  |  |  |  |
| **4** | cat urine (20 min) |  | Wet bedding |  |  |  |  |
| **5** |  |  |  |  |  |  |  |
| **6** |  |  |  |  |  |  |  |
| **7** |  |  |  |  |  |  |  |
| **8** |  |  |  |  |  |  |  |
| **9** |  |  |  |  |  |  |  |
| **10** |  |  |  |  |  |  |  |
| **11** |  |  |  |  |  |  |  |
| **12** |  |  |  |  |  |  |  |

| **July** | 1 | 2 | 3 | 4 | 5 | 6 | 7 |
| --- | --- | --- | --- | --- | --- | --- | --- |
| **Week3** | **Monday** | **Tuesday** | **Wednesday** | **Thursday** | **Friday** | **Saturday** | **Sunday** |
| **1** |  | no bedding |  |  |  | Wet bedding (200 mL water) |  |
| **2** |  |  |  |  |  |  |  |
| **3** |  |  |  |  |  |  |  |
| **4** |  |  |  |  |  |  |  |
| **5** |  |  |  |  |  |  |  |
| **6** |  |  |  |  |  |  |  |
| **7** |  |  |  |  |  |  |  |
| **8** |  |  |  |  |  | Restraint (25 minutes) |  |
| **9** |  |  | **TREATMENT BEGINS** |  |  |  |  |
| **10** |  |  |  |  |  |  |  |
| **11** |  |  |  |  |  |  |  |
| **12** |  |  |  |  |  |  |  |
| **1** |  |  |  | lights off 1.5 hours from 1 pm-2:30 pm |  | new bedding |  |
| **2** |  |  |  |  |  |  |  |
| **3** |  |  |  |  |  |  |  |
| **4** |  |  |  |  |  |  | fox urine (20 min) |
| **5** | no bedding | Reduced space + **dirty bedding** |  |  |  |  |  |
| **6** |  |  |  |  | Wet bedding (200 mL water) |  |  |
| **7** |  |  |  |  |  |  |  |
| **8** |  |  |  |  |  |  |  |
| **9** |  |  |  |  |  |  |  |
| **10** |  |  |  |  |  |  |  |
| **11** |  |  |  |  |  |  |  |
| **12** |  |  |  |  |  |  |  |

| **July** | 8 | 9 | 10 | 11 | 12 | 13 | 14 |
| --- | --- | --- | --- | --- | --- | --- | --- |
| **Week4** | **Monday** | **Tuesday** | **Wednesday** | **Thursday** | **Friday** | **Saturday** | **Sunday** |
| **1** |  |  | social stress (cage exchange) + no bedding |  |  | social stress (cage exchange) + no bedding |  |
| **2** |  |  |  |  |  |  |  |
| **3** |  |  |  |  |  |  |  |
| **4** |  |  |  |  |  |  |  |
| **5** |  |  |  |  |  |  |  |
| **6** |  |  |  |  |  |  |  |
| **7** |  |  |  |  |  |  |  |
| **8** |  |  |  |  |  |  |  |
| **9** |  |  |  | forced bath (40 min) |  |  |  |
| **10** |  |  |  |  |  |  |  |
| **11** |  |  |  |  | restraint (35 min) |  |  |
| **12** | cage tilt |  |  | new bedding |  |  |  |
| **1** |  | social stress (cage exchange) + no bedding | restraint (30 min) + new bedding |  |  |  | wet bedding + reduced space |
| **2** |  |  |  |  |  |  |  |
| **3** |  |  |  |  |  |  |  |
| **4** | cat urine (20 min) |  |  |  |  |  |  |
| **5** |  |  |  |  | social stress (2 mice rotation) |  |  |
| **6** |  |  |  |  |  |  |  |
| **7** |  |  |  |  |  |  |  |
| **8** |  |  |  |  |  |  |  |
| **9** |  |  |  |  |  |  |  |
| **10** |  |  |  |  |  |  |  |
| **11** |  |  |  |  |  |  |  |
| **12** |  |  |  |  |  |  |  |

| **July** | 15 | 16 | 17 | 18 | 19 | 20 | 21 |
| --- | --- | --- | --- | --- | --- | --- | --- |
| **Week5** | **Monday** | **Tuesday** | **Wednesday** | **Thursday** | **Friday** | **Saturday** | **Sunday** |
| **1** | wet bedding + reduced space |  |  | wet bedding |  | reduced space |  |
| **2** |  |  |  |  |  |  |  |
| **3** |  |  |  |  |  |  |  |
| **4** |  |  |  |  |  |  |  |
| **5** |  |  |  |  |  |  |  |
| **6** |  |  |  |  |  |  |  |
| **7** |  |  |  |  |  |  |  |
| **8** |  |  |  |  |  |  |  |
| **9** |  |  | fox urine (20 min) + restraint |  | **LIGHTS OFF & Social Stress: 2 mouse exchange** |  |  |
| **10** |  |  |  |  |  |  |  |
| **11** |  |  |  |  |  |  |  |
| **12** |  | forced bath (25 min) |  |  |  |  | bobcat urine (20 min) + **LIGHTS OFF** |
| **1** |  |  | wet bedding | restraint (40 min) |  |  |  |
| **2** |  |  |  | new bedding | cage tilt & reduced space |  |  |
| **3** |  | social environment stress (cage exchange) |  |  |  |  |  |
| **4** | cage tilt |  |  |  | reduced space |  |  |
| **5** |  |  |  |  |  |  |  |
| **6** |  |  |  |  |  |  |  |
| **7** |  |  |  |  |  |  |  |
| **8** |  |  |  |  |  |  |  |
| **9** |  |  |  |  |  |  |  |
| **10** |  |  |  |  |  |  |  |
| **11** |  |  |  |  |  |  |  |
| **12** |  |  |  |  |  |  |  |

| **July** | 22 | 23 | 24 | 25 | 26 | 27 | 28 |
| --- | --- | --- | --- | --- | --- | --- | --- |
| **Week6** | **Monday** | **Tuesday** | **Wednesday** | **Thursday** | **Friday** | **Saturday** | **Sunday** |
| **1** |  |  |  |  |  |  | **no bedding & space divider** |
| **2** |  |  |  |  |  |  |  |
| **3** |  |  |  |  |  |  |  |
| **4** |  |  |  |  |  |  |  |
| **5** |  |  |  |  |  |  |  |
| **6** |  |  |  |  |  |  |  |
| **7** |  |  |  |  |  |  |  |
| **8** |  |  | fox urine (20 min) + restraint |  | **Elevated Plus Maze - new bedding after behavior** |  | **Open Field** |
| **9** |  | restraint (30 min) |  | lights on/off from 9-12 |  |  |  |
| **10** |  |  | social environment stress (cage exchange) |  |  |  |  |
| **11** |  | Dark + cage tilt |  |  |  | no bedding and space divider |  |
| **12** |  |  |  |  |  |  |  |
| **1** |  |  |  |  |  |  |  |
| **2** |  |  |  |  |  |  |  |
| **3** |  |  |  |  |  |  |  |
| **4** |  |  |  |  |  |  |  |
| **5** |  |  |  |  |  |  |  |
| **6** |  |  |  |  |  |  |  |
| **7** |  |  |  |  |  |  |  |
| **8** |  |  |  |  |  |  |  |
| **9** |  |  |  |  |  |  |  |
| **10** |  |  |  |  |  |  |  |
| **11** |  |  |  |  |  |  |  |
| **12** |  |  |  |  |  |  |  |

| **July-August** | 29 | 30 | 31 | 1 | 2 | 3 | 4 |
| --- | --- | --- | --- | --- | --- | --- | --- |
| **Week7** | **Monday** | **Tuesday** | **Wednesday** | **Thursday** | **Friday** | **Saturday** | **Sunday** |
| **1** |  |  |  |  |  |  |  |
| **2** |  |  |  |  |  |  |  |
| **3** |  |  |  |  |  |  |  |
| **4** |  |  |  |  |  |  |  |
| **5** |  |  |  |  |  |  |  |
| **6** |  |  |  |  |  |  |  |
| **7** |  | **NSF** |  | **Cookie Test 1st session** |  | **Cookie Test 2nd session** |  |
| **8** |  |  |  |  | restraint (30 min) |  |  |
| **9** |  |  |  |  |  |  |  |
| **10** |  |  | bob cat urine + reduced space |  |  |  |  |
| **11** |  |  |  |  |  |  |  |
| **12** |  |  |  |  |  |  |  |
| **1** |  |  |  |  |  |  |  |
| **2** |  |  |  |  | Dark + cage tilt |  |  |
| **3** |  |  | **Social Stress: 2 mouse exchange** |  |  |  |  |
| **4** | FOOD DEPRIVE & New Cages for Group A |  |  |  |  |  |  |
| **5** |  |  |  |  |  |  |  |
| **6** |  |  |  |  |  |  |  |
| **7** |  |  |  |  |  |  |  |
| **8** |  |  |  |  |  |  |  |
| **9** |  |  |  |  |  |  |  |
| **10** |  |  |  |  |  |  |  |
| **11** |  |  |  |  |  |  |  |
| **12** |  |  |  |  |  |  |  |
